# Impact of video feedback system on medical students’ perception of their clinical performance assessment

**DOI:** 10.1186/s12909-019-1688-6

**Published:** 2019-07-08

**Authors:** Bee Sung Kam, So Jung Yune, Sang Yeoup Lee, Sun Ju Im, Sun Yong Baek

**Affiliations:** 1 Department of Medical Education, Pusan National University School of Medicine, Yangsan, 50612 South Korea; 2 Family Medicine Clinic and Research Institute of Convergence of Biomedical Science and Technology, Pusan National University Yangsan Hospital, Yangsan, South Korea

**Keywords:** Clinical skills, Assessment, Feedback, Video

## Abstract

**Background:**

Providing feedback on student performance in the clinical performance assessment (CPA) is meaningful in that it helps students understand their strengths and weaknesses. This study compared students’ perception of their CPA scores before and after providing personalized video feedback.

**Methods:**

Two identical online surveys of Year 1 medical students (*N* = 103) that had undergone CPA were conducted to evaluate students’ perceptions about their CPA scores before and after video feedback. Students were given their test scores with assessment analysis reports immediately after completing the CPA. Top-scored students from each station agreed to provide their video-recorded performance to the rest of the students.

**Results:**

After comparing their performance video and top-scored video at each station, medical students were more aware of their CPA total score, clinical performance examination (CPX) total score, score of each CPX station, section score for the CPX station, history taking section score, physical examination section score, and doctor-patient relationship section score. Moreover, students became more convinced of their own weaknesses from their history taking and patient education section after viewing video feedback than before.

**Conclusion:**

The use of the video feedback system might help students recognize their CPA results and identify their strengths and weaknesses.

**Electronic supplementary material:**

The online version of this article (10.1186/s12909-019-1688-6) contains supplementary material, which is available to authorized users.

## Background

Feedback is a critical component necessary for medical students to perform effectively and in a timely manner in clinical settings [1, 2]. However, feedback often does not satisfy both students and evaluators [3–6]. Clinical performance assessment (CPA) generally employs an analytical checklist for each station that is provided by evaluators as feedback, enabling students to recognize their strengths or weaknesses via the scores of the feedback. However, students sometimes feel that the score generated from the checklist is insufficient and dose not properly assess their performance; therefore, the evaluator provides additional feedback in several other forms, including hand-written comments, immediate verbal feedback, and briefing session. Hand-written comments in CPA can provide original, good, and sometimes powerful information [7–12]. However, because the Objective Structured Clinical Examination (OSCE) and Clinical Performance Examination (CPX) are performed in 5 or 10 min, respectively, the evaluator faces a time limit when providing feedback. Moreover, there is an additional time delay in receiving feedback cards because they are delivered to students after completion of the entire CPA; accordingly, a student might not recall the situation. Another form of feedback is immediate verbal feedback, which is very effective when the evaluator makes it prompt, precise and to the point. This type of feedback is much more effective when combined with written comments [8]. Positive verbal feedback (praise) may cheer up a student, while immediate verbal feedback itself might enhance student anxiety and cause them to perform negatively in subsequent tasks. In the worst scenarios, students might lose control of their emotional behavior and receive lower scores from the remaining stations [13–15]. A third feedback method involves gathering all of the students into the classroom, briefing them on the overall CPA results, and pointing out the most common errors that students have made. This is timely and effective but does not provide individual feedback.

Good feedback makes students engage in the feedback process rather than the technical aspect of the feedback [4]. Moreover, good feedback requires that the student’s performance be carefully monitored [16, 17]. Therefore, Keele University School of Medicine has developed a personalized audio feedback tool that uses a hand-held digital mp3 player to improve the OSCE performance of students [18]. Although this method is convenient and acceptable to both students and evaluators, it may be somewhat difficult to grasp the meaning of the comments because the performance situation or the illustration in which the comment in the audio file is given cannot be seen [19–23]. Therefore, we recently developed an individualized video feedback system in addition to the online-written comments we already employed in the CPA to provide students with more feedback regarding self-learning. Providing effective feedback to medical students corresponds with the shift toward learner-centered education from teacher-centered education. We gave feedback via hand-written comments before implanting video feedback. Although this allowed teachers to feel relieved that they have provided feedback, but they did not know at whether students were receiving the feedback or the intention of teachers [24]. Similarly, like teaching and learning, the evaluator gives a feedback, and if the feedback is not accepted by those being evaluated, the feedback might be useless to the student. This study is one of a series of feedback studies, in which students’ perceptions of the evaluator’s feedback was investigated. Students may accept that the score generated from a checklist properly assessed for their performance if personalized video feedback of their performance during CPA is provided in addition to hand-written comments and an analytical checklist. Therefore, in the present study, we compared students’ perception of their score results before and after providing personalized video feedback to develop a more effective feedback method that can be applied in CPA situations of medical education.

## Methods

### Study participants and design

**Fig. 1:**
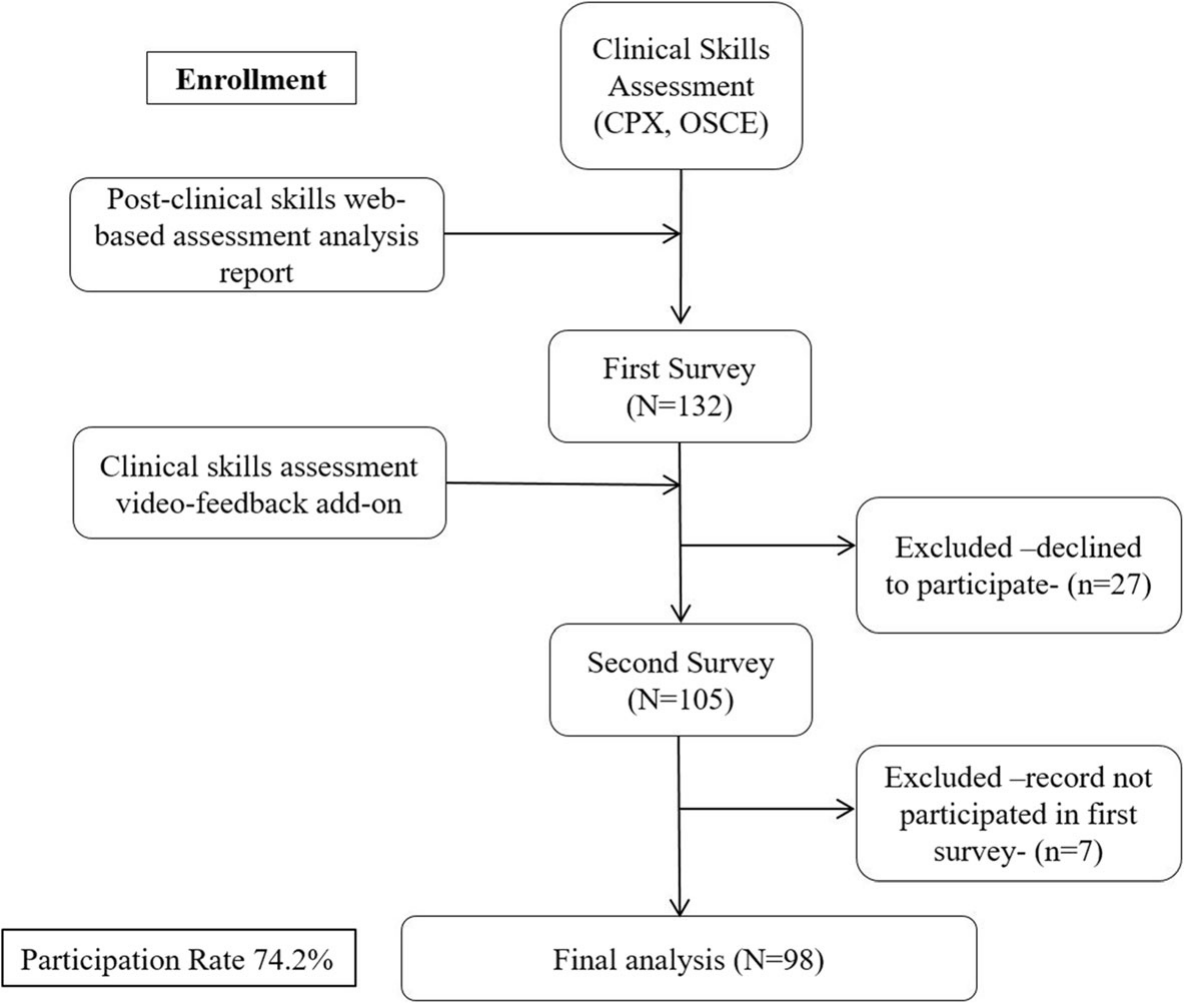
Study flowchart

A questionnaire-based before and after study was used to survey first year medical students of Pusan National University School of Medicine in the second semester of 2012. This study was reviewed and given exempt status by the Institutional Review Board of Pusan National University Yangsan Hospital (IRB No. 05–2017-102). Because we analyzed data retrospectively and anonymously by assigning each subject a distinct number, the institutional review board did not require informed consent from participants. A total of 131 first year medical students underwent CPA including CPX and OSCE. Immediately after completing the CPA, students were given their test scores with a computer assisted assessment analysis report. The top-scored students from each station agreed to provide their video-recorded performance to the rest of the students. Basically, all students received their own video-recorded performance. In addition, videos of the best students of each station were provided to the rest of the students. This video feedback system was designed to allow students to compare the recorded video of the best student at each station with their own video-recorded performance so they could realize their strengths and weaknesses. Two identical online surveys were conducted to evaluate students’ perceptions of their CPA scores before and after the video feedback. We developed a program so that only students who responded to the first questionnaire were allowed to view their own video, followed by the recorded video of the best student, after which the students were allowed to respond to the second questionnaire. A total of 131 students answered the first questionnaire, while only 103 (78.6%) responded to the second questionnaire (Fig. 1). The questionnaire was developed based on an extensive review of the literature [23, 25–27] and the consensus of five faculty members in the department of medical education and 20 faculty members of Clinical Skills Committee, who were expert educators and clinical teachers. Students were unaware of the first and second survey questions before receiving the corresponding feedback.

### Clinical performance assessment

All students completed the CPA, which was composed of three CPX stations and three OSCE stations. Cases were selected to represent common acute conditions, chronic conditions, and counseling cases. The three CPX stations were as follows: acute abdominal pain, headache, and delivering bad news. The three OSCE stations included basic clinical skills such as muscular injection, burn dressing, and cranial nerve examination. In the CPX, Standard Patients (SPs) presented a variety of patient problems.

Each skills station was equipped with a computer assisted assessment system as an instrument for conducting the CPA. Evaluators evaluated the performance of each student and filled out an in-depth station specific online checklist. After assessing the performance of a student, evaluators added online-written comments about the main weak points. All station encounters were digitally recorded using a room equipped with a microphone and a camera encoded with H.264 standard compression. After the entire class had completed their assessment, students received a report indicating their scores for each section (history taking, physical exam, counseling and communication skills) and overall of the cases. The evaluator’s online-written comments from each station were provided to students to improve their self-directed learning skills. Students who did not attain a passing score at each station were shown a “FAIL” mark for that station, and if overall scores for all stations were below a passing range, an overall “FAIL” mark was shown for the overall assessment. Individualized feedback including their scores (pass, fail, rank, minimum, maximum, total score, standard deviation), the top-score of the best student at each station, and online-written comments were provided to CPA applicants before performing the first survey. A retake of an examination was permitted for students with the scores 1 SD below the mean. A pass/fail decision for CPA is based on the total scores 2 SD below from the mean. On average, no more than three students failed to pass this assessment.

### Materials

For the development of the questionnaire, two rounds of Delphi expert consultation were conducted with five faculty members in the department of medical education and a faculty focus group (*n* = 10) selected among members of the Clinical Skills Committee. During the first round, experts were asked to provide their opinions in a questionnaire consisting of open-ended questions about the evaluation area and evaluation items. Items selected in the first-round analysis were presented to each expert by email in a second round, when experts were asked to use the Likert 5-point scale to evaluate whether they agreed with inclusion or exclusion of items according to the importance of each factor and item. Experts were also asked to describe the suitability of the evaluation system and the comments pertaining to the items to be revised and supplemented by the evaluation factors. Experts did not meet face-to-face, and they completed their assessments independently. The content validity was based on the content validity ratio (CVR) proposed by Lawshe [28]. The CVR ranged from a maximum of + 1.0 to a minimum of − 1.0. If the CVR was positive, more than half of the respondents answered ‘appropriate’, which meant they were rated 4 or 5 on the Likert 5-point scale. The CVR gives the minimum value according to the number of panels. When the value was above the minimum value, it is judged that there is content validity for the item. The number of panels in this study was 15, and the content validity was found to be more than 0.49. In the second round, all of the developed items were available because the average of the validity responses was 4.5 or more. Finally, the questionnaire consisted of 4 items regarding CPA total score reports including CPX and OSCE, 12 items regarding CPX score reports, 2 items regarding OSCE score reports, 2 items regarding online written comments, and 2 items regarding video feedback system. The contents of the questions are shown in Table 1 (Additional file 1). The same questionnaire was administered before and after providing video feedback. Only questions pertaining to the usefulness of video feedback were added. Answers were given on a 5-point Likert-type scale from strongly disagree to strongly agree, which is used to allow the individual to express how much they agree or disagree with each question. Two open-ended questions concerning the CPX station that students disagreed with for the CPX station score and OSCE station score were presented at the end of the questionnaire. Completion of the questionnaire took approximately 30 min.

**Table 1:** Effects of video feedback system on students’ perceptions regarding their clinical performance assessment (*N* = 103)

	Before^a,b^	After^a,b^	*P* value	Cohen’s d
Regarding CPA total score reports including CPX and OSCE				
Do you agree with your total CPA score?	3.80 ± 0.62	3.91 ± 0.78	0.057	0.16
Do you agree with your total CPX score?	3.75 ± 0.62	3.93 ± 0.76	0.011	0.26
Do you agree with your total OSCE score?	3.76 ± 0.79	3.88 ± 0.80	0.150	0.15
Can you perceive your weak points from your total CPX and OSCE scores?	3.91 ± 0.77	3.95 ± 0.63	0.717	0.06
Regarding CPX score reports				
Do you agree with your each CPX station score?	3.82 ± 0.74	3.99 ± 0.73	0.033	0.23
Can you perceive your weak points from each CPX station score?	3.87 ± 0.76	3.92 ± 0.82	0.621	0.06
Do you agree with your CPX station section score?	3.83 ± 0.62	3.99 ± 0.69	0.017	0.24
Can you perceive your weak points from your CPX station section score?	3.91 ± 0.63	4.00 ± 0.73	0.307	0.13
Do you agree with your history taking section score?	3.89 ± 0.64	4.02 ± 0.78	0.080	0.18
Can you perceive your weak points from your history taking section score?	3.88 ± 0.68	4.03 ± 0.79	0.096	0.20
Do you agree with your physical examination section score?	3.87 ± 0.61	4.06 ± 0.75	0.016	0.28
Can you perceive your weak points from your physical examination section score?	3.96 ± 0.67	4.06 ± 0.76	0.283	0.14
Do you agree with your patient education section score?	3.88 ± 0.58	3.99 ± 0.75	0.132	0.16
Can you perceive your weak points from your patient education section score?	3.81 ± 0.81	4.09 ± 0.74	0.003	0.36
Do you agree with your doctor-patient relationship section score?	3.84 ± 0.59	4.03 ± 0.77	0.007	0.28
Can you perceive your weak points from your doctor-patient relationship section score?	3.89 ± 0.68	4.00 ± 0.74	0.223	0.17
Regarding OSCE score reports				
Do you agree with each OSCE station score?	3.92 ± 0.59	4.00 ± 0.80	0.341	0.11
Can you perceive your weak points from each OSCE station score?	3.99 ± 0.66	4.02 ± 0.74	0.724	0.04
Regarding online-written comments				
Do you agree with feedback in online-written comments you received for each station?	4.05 ± 0.63	4.11 ± 0.82	0.505	0.08
Can you perceive your weak points from online-written comments?	3.90 ± 0.72	4.06 ± 0.87	0.152	0.20
Regarding video feedback system				
Was it helpful to review your own videos?		4.03 ± 0.80		
Was it helpful to review the best student’s video for each station?		4.25 ± 0.78		

*CPA* clinical performance assessment, *CPX* clinical performance examination, *OSCE* objective structured clinical examination

Note: ^a^The extent to which students agree/disagree with the following questions

^b^5-Likert scale: strongly agree, agree, undecided, disagree, and strongly disagree

### Statistical analysis

Descriptive statistics were used to characterize and describe the sample features. For comparisons of differences in students’ perceptions before and after providing video feedback, a paired *t*-test was used. Effect sizes were calculated using Cohen’s d with small, medium and large effects having the values of 0.0–0.2, greater than 0.2 to 0.5 and above 0.5, respectively [29]. Students’ perceptions regarding their own score after the total CPA score report, online-written comment, and video feedback were compared using ANOVA. The level of significance was set at 0.05 and statistical analyses were conducted using SPSS 13.0 for Windows (SPSS Inc., Chicago, IL, USA).

## Results

Table 1 shows the difference in agreement and perception of students regarding their scores before and after receiving video feedback. For all questions, students’ perception was higher after viewing video feedback than before. After comparing the performance video and top-scored video for each station, medical students were more aware of their CPX total score (*P* = 0.011), each CPX station score (*P* = 0.033), CPX station section score (*P* = 0.017), physical examination section score (*P* = 0.016), and doctor-patient relationship section score (*P* = 0.007, Table 1). Students agreed to the total scores for the CPA and history taking section score better after viewing video feedback than before. Students were also better able to perceive their own weaknesses from the history taking section score (*P* = 0.096) and patient education section score (*P* = 0.003) after viewing video feedback than before. However, despite providing video feedback to students, there was no difference in other agreement and perception from the students’ perspective. Tables 2 and 3 show changes in the perception of students who did not agree with their own CPX station score before and after video feedback. Whether or not students agreed to agree on their scores before video feedback, most students accepted their scores after the video feedback. On the contrary, although very few, some students initially accepted their scores, but were not convinced after the video feedback. Overall, students assessed the usefulness of video feedback (4.25 ± 0.78) higher than that of the computer assisted assessment analysis report (3.80 ± 0.62) or online-written comment (3.92 ± 0.59).

**Table 2:** Number of students who did not agree with their own CPX station score (*N* = 103)

CPX station	Before	After	
Acute abdominal pain	5	2	Partly resolved
Headache	1	–	Resolved
Delivering bad news	2	–	Resolved
Acute abdominal pain		1	New
Delivering bad news		2	New
Total	8	5	

*CPX* clinical performance examination

**Table 3:** Number of students who did not agree with their own OSCE station score (*N* = 103)

OSCE station	Before	After	
Muscular Injection	2	–	Resolved
Burn Dressing	2	1	Partly resolved
Cranial nerve examination	3	1	Almost resolved
Cranial nerve examination		1	New
Total	7	3	

*OSCE* objective structured clinical examination

## Discussion

This study was conducted to evaluate the effects of providing personalized video feedback of first year medical student’s performance during CPA in addition to hand-written comments on the way they perceive their score results from an analytical checklist. The developed method was designed to allow students to compare the recorded video of the best student at each station with the recorded video of the exam they were performing so they could realize what they did well and what skills they lacked. The results of the present study showed that students were more likely to agree with the analytical checklist score of their CPA after they compared the recorded video of the best student at each station with the recorded video of the exam they performed. The video feedback allowed them to realize what they did well and what skills they lacked [30]. In addition, they were more likely to accept their CPA total score, CPX total score, each CPX station score, history taking section score, physical examination section score, and doctor-patient relationship section after receiving video feedback. The satisfaction rate of the video feedback system was more than 4 out of 5. This change could be regarded as meaningful and indicates that the intervention of the video feedback seemed to have an effect on how students perceived their performance; however, care should be taken when interpreting these results. In addition, eight students (7.77%) did not agree with their CPX station score, but after video feedback, only 4.85% did not agree. Moreover, seven students (6.80%) disagreed with their OSCE station score before seeing the video, but this dropped to 3.91% after receiving the video feedback. Although more students agreed with their online-written comments after receiving video feedback, this difference was not statistically significant. Even if students complete a station assessment in less than the allotted time, it is still time-consuming for the evaluator to provide hand-written comments to the students. As a result, some critical comments may be eliminated if too many applicants are evaluated within a given time frame.

Based on these findings, video feedback was more effective than analytic checklist score or online-written comments at helping students understand CPA outcomes. In addition, the video feedback system used in this study appeared to be an improved form in that it made it possible to identify the performance situation, which was the limitation of the mp3 audio feedback tool introduced at Keele University School of Medicine. In previous studies, the video feedback system was very useful in that it could check the performance of the recorded video and provide feedback [31]. Lindon-Morris and Laidlaw [32] reported that student’s perceived their self-awareness to be unfavorable to their performance in the presence of the video camera, but that they could compare their videos with those of other students to monitor their performance more accurately and refer to their students’ communication strategies to modify their own communication strategies during clinical communication training using technology including video feedback. In a previous study in the field of nursing, video-feedback showed changes in communication, clinical competence and motivational interviewing skills of prospective nurses [33]. In addition, the experimental group that received video feedback had higher scores for knowledge, performance competence of core basic nursing skills, self-efficacy, learning motivation, and learning satisfaction than control groups that did not receive video feedback in previous studies [34, 35].

It should be noted that video feedback does not always have a positive effect, and that it can produce different learning effects depending on how it is provided to the learner. Specifically, video feedback should be provided to learners in combination with other additional methods to generate positive learning effects [36]. In addition, attention-focusing cues should be given before the video is presented and combined with error-correction information to provide the learner with the information [37]. It would also be helpful to combine other feedback methods with videos of professional models for use as templates for comparison to one’s own videos to detect errors [38]. In this study, changes in student perception in OSCE as a result of video feedback were not statistically significant. Accordingly, it is necessary to carefully consider how to provide video feedback. Even though it was a very small percentage, some students agreed to their CPX scores of ‘acute abdominal pain’ and ‘delivering bad news’ sections before viewing the video feedback, but after viewing the video feedback, they did not accept their score unlike our expectations. The advantage of the video feedback system developed in this study is that it enabled students to compare their performance with that of the best students, which allowed them to recognize the reasons for their CPA results, develop their strengths, and complement their weaknesses. However, although the video feedback system used in this study allowed learners to see their own strengths and weaknesses in the previous examination, it did not include direct feedback on error corrections or regarding what to do in the next examination. Moreover, the results of video-feedback could not be acknowledged because online-written feedback and video feedback were presented to students separately in binary form. Therefore, it will be necessary to address this issue in the future to enable continued development of the video feedback system. Also, effectiveness of the video feedback system for improving clinical performance, stakeholder feedback for successful video feedback systems, or comparison among different feedback systems need to be conducted in the future.

It should be noted that this study was limited in that acceptance of the test score was part of the overall feedback system acceptance, which may not be sufficient alone, because this is an indirect measure that requires more caution when interpreting the results. Feedback might be useful if it is accepted by those being evaluated. However, the results of this study revealed some students who, although accepting their scores at first, no longer accepting them viewing the video feedback. Accordingly, additional interviews should be conducted to ensure that students understood their scores well and considered the test results to be fair and appropriate; unfortunately, however, such interviews were outside the scope of this study.

## Conclusions

In summary, the results of this study suggest that the use of a video feedback system in CPA of medical education can help students recognize their CPA results and identify their strengths and weaknesses. Future studies should include development of a video feedback system that complements the educational usefulness derived from the results of this study so that it can be used more actively in medical education. Additionally, a more realistic and direct personalized feedback system needs to be introduced into clinical skill education in the future.

## Additional file

Additional file 1: The questionnaire used in the present study. Questionnaire S1. Students’ perceptions regarding their clinical performance assessment before viewing video feedback. Questionnaire S2. Students’ perceptions regarding their clinical performance assessment after viewing video feedback. (DOCX 21 kb)

## Data Availability

The datasets used and analyzed during the current study are available from the corresponding author on reasonable request.

## Abbreviations

CPA: Clinical performance assessment
CPX: Clinical performance examination
OSCE: Objective structured clinical examination
