# Supplementary material for: Impact of video feedback system on medical students’ perception of their clinical performance assessment
Source: BMC Med Educ. 2019 Jul 8;19:252. doi: 10.1186/s12909-019-1688-6 (PMC6615181; doi:10.1186/s12909-019-1688-6)
Supplement: Supplementary file 1 — The questionnaire used in the present study. Questionnaire S1. Students’ perceptions regarding their clinical performance assessment before viewing video feedback. Questionnaire S2. Students’ perceptions regarding their clinical performance assessment after viewing video feedback. (DOCX 21 kb) [file 12909_2019_1688_MOESM1_ESM.docx]

Questionnaire 1. Students’ perceptions regarding their clinical performance assessment before viewing video feedback.

To what extent do you agree or disagree with the following statement

| **5-Likert scale** | strongly agree | agree | undecided | disagree | strongly disagree |
| --- | --- | --- | --- | --- | --- |
| **1. Regarding CPA total score reports including CPX and OSCE** | | | | |  |
| Do you agree with your total CPA score? |  |  |  |  |  |
| Do you agree with your total CPX score? |  |  |  |  |  |
| Do you agree with your total OSCE score? |  |  |  |  |  |
| Can you perceive your weak points from your total CPX and OSCE scores? |  |  |  |  |  |
| **2. Regarding CPX score reports** | | | | |  |
| Do you agree with your each CPX station score? |  |  |  |  |  |
| Can you perceive your weak points from each CPX station score? |  |  |  |  |  |
| Do you agree with your CPX station section score? |  |  |  |  |  |
| Can you perceive your weak points from your CPX station section score? |  |  |  |  |  |
| Do you agree with your history taking section score? |  |  |  |  |  |
| Can you perceive your weak points from your history taking section score? |  |  |  |  |  |
| Do you agree with your physical examination section score? |  |  |  |  |  |
| Can you perceive your weak points from your physical examination section score? |  |  |  |  |  |
| Do you agree with your patient education section score? |  |  |  |  |  |
| Can you perceive your weak points from your patient education section score? |  |  |  |  |  |
| Do you agree with your doctor-patient relationship section score? |  |  |  |  |  |
| Can you perceive your weak points from your doctor-patient relationship section score? |  |  |  |  |  |
| **3. Regarding OSCE score reports** | | | | |  |
| Do you agree with each OSCE station score? |  |  |  |  |  |
| Can you perceive your weak points from each OSCE station score? |  |  |  |  |  |
| **4. Regarding online-written comments** | | | | |  |
| Do you agree with feedback in online-written comments you received for each station? |  |  |  |  |  |
| Can you perceive your weak points from online-written comments? |  |  |  |  |  |

CPA, clinical performance assessment; CPX, clinical performance examination; OSCE, objective structured clinical examination

**5. Describe CPX stations you did not agree with their own CPX station score?**

Questionnaire 2. Students’ perceptions regarding their clinical performance assessment after viewing video feedback.

To what extent do you agree or disagree with the following statement

| **5-Likert scale** | strongly agree | agree | undecided | disagree | strongly disagree |
| --- | --- | --- | --- | --- | --- |
| **1. Regarding CPA total score reports including CPX and OSCE** | | | | |  |
| Do you agree with your total CPA score? |  |  |  |  |  |
| Do you agree with your total CPX score? |  |  |  |  |  |
| Do you agree with your total OSCE score? |  |  |  |  |  |
| Can you perceive your weak points from your total CPX and OSCE scores? |  |  |  |  |  |
| **2. Regarding CPX score reports** | | | | |  |
| Do you agree with your each CPX station score? |  |  |  |  |  |
| Can you perceive your weak points from each CPX station score? |  |  |  |  |  |
| Do you agree with your CPX station section score? |  |  |  |  |  |
| Can you perceive your weak points from your CPX station section score? |  |  |  |  |  |
| Do you agree with your history taking section score? |  |  |  |  |  |
| Can you perceive your weak points from your history taking section score? |  |  |  |  |  |
| Do you agree with your physical examination section score? |  |  |  |  |  |
| Can you perceive your weak points from your physical examination section score? |  |  |  |  |  |
| Do you agree with your patient education section score? |  |  |  |  |  |
| Can you perceive your weak points from your patient education section score? |  |  |  |  |  |
| Do you agree with your doctor-patient relationship section score? |  |  |  |  |  |
| Can you perceive your weak points from your doctor-patient relationship section score? |  |  |  |  |  |
| **3. Regarding OSCE score reports** | | | | |  |
| Do you agree with each OSCE station score? |  |  |  |  |  |
| Can you perceive your weak points from each OSCE station score? |  |  |  |  |  |
| **4. Regarding online-written comments** | | | | |  |
| Do you agree with feedback in online-written comments you received for each station? |  |  |  |  |  |
| Can you perceive your weak points from online-written comments? |  |  |  |  |  |

CPA, clinical performance assessment; CPX, clinical performance examination; OSCE, objective structured clinical examination

**5. Describe CPX stations you did not agree with their own CPX station score?**

**6. Regarding video feedback system**

Was it helpful to review your own videos?

|  |
| --- |

Was it helpful to review the best student’s video for each station?

|  |
| --- |
